# Severe acute Hypertriglyceridemia induced Pancreatitis treated with Insulin therapy

**DOI:** 10.51894/001c.144644

**Published:** 2025-09-30

**Authors:** Muhammad Mustafa A. Siddiqui

**Affiliations:** 1 Department of Internal Medicine Detroit Medical Center-Sinai Grace Hospital

## 123

### INTRODUCTION

Hypertriglyceridemia-induced pancreatitis causes 1 to 35 percent of all cases of acute pancreatitis. The severity of hypertriglyceridemia induced pancreatitis progressively increases when serum triglycerides are greater than 1000. Worrisome features of pancreatitis includes hypocalcemia, lactic acidosis or if a patient exhibits two or more features of systemic inflammatory response syndrome.

### CASE DESCRIPTION

This case highlights a novel presentation of hypertriglyceridemia induced pancreatitis with triglyceride levels above 2500mg/dl which was treated with insulin therapy without plasmapheresis. This case emphasizes the importance of insulin therapy, which is especially useful for healthcare systems which may be devoid of plasmapheresis. A 41 year old male with a known history of obesity, insulin dependant type 2 diabetes mellitus, recurrent pancreatitis and hypertriglyceridemia presented with acute onset epigastric pain associated. He was tachycardic and febrile on presentation and had Serum Triglyceride 2549, Lipase of 1095u/L, Lactate 3.5, hypocalcemia of 5.8 and CT abdomen and pelvis which confirmed acute interstitial edematous pancreatitis. Criteria for worrisome features of pancreatitis were met which warranted plasmapheresis therapy, it was not initiated due to unavailability. He was started on high intensity statin therapy, gemfibrozil and insulin drip with plan to transfer to another facility if triglyceride therapy remained suboptimal. Serum triglyceride levels continued to trend down with decreasing requirement of insulin which was eventually transitioned to subcutaneous insulin. Triglyceride levels plateaued in the 400s with the patient’s symptoms improving and was able to tolerate soft diet before discharge.

### DISCUSSION

The management of hypertriglyceridemia induced pancreatitis includes treatment of the acute pancreatitis as well as the reduction in serum triglyceride levels, insulin therapy and plasmapheresis. Insulin therapy is widely available and can be initiated early on during the course of the disease. There is no definitive RCT evidence for plasmapheresis available and it’s effectiveness relies highly on initiating treatment during the early stages of the disease. Despite early initiation of plasmapheresis, mortality benefit is still unclear as it has not been demonstrated in literature. This case underscores the significance of appropriate insulin therapy which may be sufficient in treating even acute severe hypertriglyceridemia induced pancreatitis despite indications being present for plasmapheresis.

